# Nanoceria seed priming enhanced salt tolerance in rapeseed through modulating ROS homeostasis and α-amylase activities

**DOI:** 10.1186/s12951-021-01026-9

**Published:** 2021-09-16

**Authors:** Mohammad Nauman Khan, Yanhui Li, Zaid Khan, Linlin Chen, Jiahao Liu, Jin Hu, Honghong Wu, Zhaohu Li

**Affiliations:** 1 MOA Key Laboratory of Crop Ecophysiology and Farming System in the Middle Reaches of the Yangtze River, College of Plant Science and Technology, Huazhong Agricultural University, Wuhan, 430070 China; 2 School of Agriculture and Technology, China Agricultural University, Beijing, 100083 China; 3 Shenzhen Institute of Nutrition and Health, Huazhong Agricultural University, Shenzhen, China; 4 Shenzhen Branch, Guangdong Laboratory for Lingnan Modern Agriculture, Genome Analysis Laboratory of the Ministry of Agriculture, Agricultural Genomics Institute at Shenzhen, Chinese Academy of Agricultural Sciences, Shenzhen, China

**Keywords:** Nanoceria, Salt stress, Seed priming, ROS homeostasis, α-amylase activity, Na^+^/K^+^ ratio

## Abstract

**Background:**

Salinity is a big threat to agriculture by limiting crop production. Nanopriming (seed priming with nanomaterials) is an emerged approach to improve plant stress tolerance; however, our knowledge about the underlying mechanisms is limited.

**Results:**

Herein, we used cerium oxide nanoparticles (nanoceria) to prime rapeseeds and investigated the possible mechanisms behind nanoceria improved rapeseed salt tolerance. We synthesized and characterized polyacrylic acid coated nanoceria (PNC, 8.5 ± 0.2 nm, −43.3 ± 6.3 mV) and monitored its distribution in different tissues of the seed during the imbibition period (1, 3, 8 h priming). Our results showed that compared with the no nanoparticle control, PNC nanopriming improved germination rate (12%) and biomass (41%) in rapeseeds (*Brassica napus*) under salt stress (200 mM NaCl). During the priming hours, PNC were located mostly in the seed coat, nevertheless the intensity of PNC in cotyledon and radicle was increased alongside with the increase of priming hours. During the priming hours, the amount of the absorbed water (52%, 14%, 12% increase at 1, 3, 8 h priming, respectively) and the activities of α-amylase were significantly higher (175%, 309%, 295% increase at 1, 3, 8 h priming, respectively) in PNC treatment than the control. PNC primed rapeseeds showed significantly lower content of MDA, H_2_O_2_, and ^•^O_2_^−^ in both shoot and root than the control under salt stress. Also, under salt stress, PNC nanopriming enabled significantly higher K^+^ retention (29%) and significantly lower Na^+^ accumulation (18.5%) and Na^+^/K^+^ ratio (37%) than the control.

**Conclusions:**

Our results suggested that besides the more absorbed water and higher α-amylase activities, PNC nanopriming improves salt tolerance in rapeseeds through alleviating oxidative damage and maintaining Na^+^/K^+^ ratio. It adds more knowledge regarding the mechanisms underlying nanopriming improved plant salt tolerance.

**Graphical abstract:**

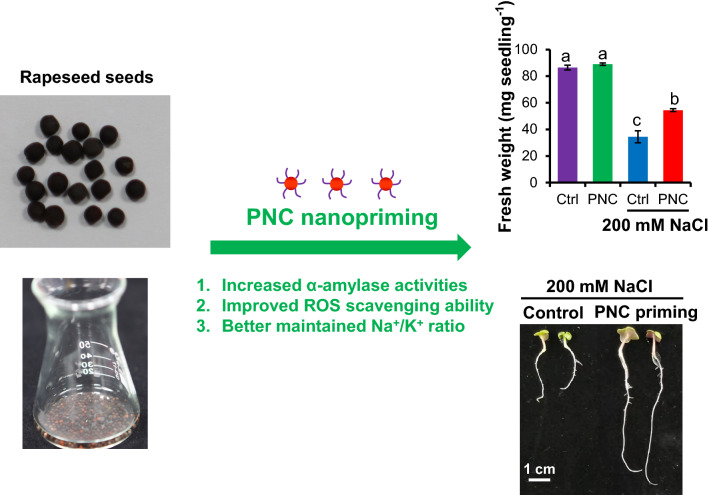

**Supplementary Information:**

The online version contains supplementary material available at 10.1186/s12951-021-01026-9.

## Background

Rapeseed (*Brassica napus* L.) is one of the important oilseed crops in the world [1]. In the past decade, the *Brassica napus* total harvested area, productivity, and annual grain yield increased by 17.6% (29.5–34.7 million ha), 50.3% (50.6–76.1 million tons), and 27.8% (1716–2194 kg ha^−1^) respectively, reflecting that the demand for *Brassica napus* is continuing to increase [2]. However, stresses such as drought, salinity, and heat limit rapeseed production [3]. To date, about 20% (~ 953 million ha) of the total global land is salt-affected [3]. Salinity is one of the major stresses impairing seed germination ability or resulting longer germination time [4, 5]. Besides seed germination, salinity stress affects the performance of *Brassica napus* plants in early seedling growth stage, inhibits photosynthetic functions in vegetative growth stage, and reduces seed production [2, 6].

Seed germination is the first step of establishing a plant. The process of seed germination is categorized into three subsequent phases: (1) imbibition phase (water absorption), (2) lag phase (reserves metabolism), and (3) radicle protrusion [7]. To improve seed germination under hostile conditions, many techniques have been applied in agricultural practice. Seed priming is one of the widely adopted techniques. Seed priming results in the emergence of seedlings through the regulation of metabolic processes during germination phase under stress conditions [8]. Seed priming ensures speedy and uniform germination while reduces the imbibition time [9, 10], activates pre-germinative enzymes, increases the production of metabolites [11], regulation of osmosis, and hence repairing the damaged DNA [12]. Seed priming enhances antioxidant enzymes activities and reduces lipid peroxidation [10]. Besides priming for disease control [13] and nutrient supply [14], seeds can be also imbibed in a solution containing certain solutes such as salt [15], mannitol [16], and PEG [10] etc., for the activation of pre-germinative metabolism while not allowing the seed to the fully germination stage [1]. Among the priming techniques, seed nanopriming which uses nanomaterials to prime seeds, has been reported to be successfully applied for improving germination and stress tolerance of seedlings [17]. Nanoparticles (NPs) such as AgNPs and Fe_2_O_3_ were documented to improve seed development by enhancing starch metabolism, modulating hormonal balance and triggering Fe acquisition in wheat and rice [18, 19]. Seed priming in lupine (*Lupinus albus*) with ZnO NPs played an important role in salt stress tolerance via enhancing antioxidant enzyme activities and reducing Na^+^ over-accumulation [20].

Cerium oxide nanoparticles (nanoceria, CeO_2_ NPs), due to their unique catalytic ROS (reactive oxygen species) scavenging properties, are widely used in industry, medical research and plant science [21, 22]. Cerium oxide nanoparticles were reported to reduce over-accumulated ROS, thus improving plant tolerance to stress such as salinity [23, 24], light, and temperature stress [24]. Further studies showed that after scavenging of ROS, nanoceria could modulate the activities of channel proteins related to K^+^ efflux to enable better mesophyll K^+^ retention, and upregulate the expression of *HKT1* gene to allow better shoot Na^+^ exclusion, thus improving plant salt tolerance [25, 26]. To our surprise, to date, no polyacrylic acid coated nanoceria (PNC) nanopriming was tested on rapeseeds. Whether PNC nanopriming would improve rapeseed salt tolerance is still unknown. Also, the mechanisms underlying PNC nanopriming improved plant salt tolerance are ambiguous. Our previous study showed that under paperoll condition, PNC nanopriming improved salt tolerance in cotton seedlings by modulating ROS homeostasis and Ca^2+^ signalling [27]. However, whether the underlying mechanisms such as nanoparticle distribution in PNC primed seeds, and the maintenance of ROS homeostasis and Na^+^/K^+^ ratio in the consequent established seedlings will be different between rapeseed and other crops e.g. cotton are still unknown. In germinating seeds, the enzymes responsible for the breakdown of starch are classified as α-amylase and β-amylase [28]. However, during germination, α-amylase is the predominant enzyme synthesized which mobilizes starch in the endosperm [28]. α-amylase hydrolyzes a-1,4-glucan linkages in the starch polymers [29], converting amylose to maltose and glucose (soluble sugars). The production of soluble sugars has positive relationship with seed germination [11]. The increase in soluble sugar concentration enhanced plant tolerance to several abiotic stresses such as cold, drought and salt [30]. However, to date, the role of PNC nanopriming on the relative gene expression level and activities of α-amylase and thus the soluble sugar content in seeds during the priming hours was largely overlooked. In the present study, we will try to address above questions to investigate the mechanisms regarding PNC nanopriming improved plant salt tolerance.

In this work, we studied the distribution of PNC in seed coat, cotyledon, and radicle during the priming hours. We analyzed the activities and relative gene expression of α-amylase in rapeseeds with PNC nanopriming. Furthermore, after priming, we compared the activities of antioxidant enzymes and the ROS level between PNC nanoprimed rapeseeds and the control one under salinity stress. We also investigated the Na^+^ content, K^+^ content, and Na^+^/K^+^ ratio in shoot and root in salt (200 mM NaCl) stressed *Brassica napus* plants with or without PNC nanopriming. Our results add more knowledge to nanopriming improved plant salt tolerance.

## Materials and methods

### Synthesis and characterization of nanoceria (PNC)

Following our previous paper [31], 1.08 g of cerium (III) nitrate (Sigma Aldrich, 99%) and 4.5 g poly (acrylic) acid (1800 MW, Sigma Aldrich) were dissolved in 2.5 mL and 5 mL ddH_2_O in a 50 mL conical tube, respectively. These solutions were thoroughly mixed by using a vortex mixture at 2,000 rpm for 15 min. To a 50 mL glass beaker, 15 mL of 30% ammonium hydroxide solution (Sigma Aldrich) (7.2 M) was added. The mixture of cerium (III) nitrate and poly (acrylic) acid was added dropwise into the ammonium hydroxide solution while stirring at 500 rpm overnight at room temperature in a fume hood. After 24 h, to remove any possible debris and large agglomerates, the final solution obtained was transferred to 50 mL conical tube and centrifuged at 4000×*g* for 1 h. The obtained supernatant was transferred into three 15 mL 10 kDa filters (MWCO 10 K, Millipore Inc.) and centrifuged at 4500 rpm for six cycles (45 min each cycle) for further purification. The freshly synthesized PNC solution was stored at 4 °C until further use. The final concentration of the synthesized PNC solution was calculated by recording the absorbance at 271 nm using an UV–Vis spectrophotometer according to Beer-Lambert's law (Additional file 1: Figure S1). The zeta potential and size of PNC dispersed in DI water were measured by dynamic light scattering instrument (Malvern Zetasizer, Nano). For TEM (transmission electron microscopy) imaging, 0.45 mM PNC nanoparticles were dispersed in ethanol. TEM samples were mounted on pure C grids, 200 mesh Cu (01,840, Ted Pella Inc.). FEI Talos microscope operating at 300 kV was used for recording transmission electron microscopy (TEM) images. Three independent samples were used.

### Seed materials, seed priming, stress treatments, and growth conditions

Seeds of salt-sensitive *Brassica napus* variety “Zhongshuang 11” (ZS 11) were used in this experiment [32]. After concentration screening experiment (Additional file 1: Figure S2), 0.1 mM PNC was used as a priming agent in this experiment. For pH maintenance, PNC in 10 mM TES buffer (pH 7.5 adjusted by HCl) [31] were used. 10 mM TES buffer alone was used as a control group. Seeds were immersed in PNC + TES or TES solution. The conical flasks containing the seeds and priming solution were put on a mechanical shaker (50 rpm) under dark conditions with constant gentle agitation for 8 h, and the seed to solution ratio was 1:5 (w/v) [1]. After 8 h priming, the seeds were rinsed with DI water and the seeds were kept in dark at ambient room temperature to dry-back. The primed seeds were sown in polyethylene boxes (12 × 12 × 6 cm length, breadth, and height, respectively). According to the protocols described by Khan et al. [10], the boxes contained three sterilized germination papers moistened with 10 mL of 200 mM NaCl solution or DI water. Every second day the bottom two germination papers were replaced with two new papers and 7 mL of salt solution or DI water was added to the corresponding boxes. The boxes were exposed to 14 h light (200 μmol m^−2^ s^−1^) and 10 h dark duration with 25 ± 1 and 20 ± 1 °C, respectively. The germination rate was recorded on daily basis and the germination trial was terminated 7 days after sowing. Then, the biomass was recorded immediately.

### Localization of nanoceria in *Brassica napus* seeds

To visualize the localization of nanoceria in the different tissues of seed, i.e. seed coat, cotyledon, and radicle, during priming hours, PNC were labeled with 1,1′-dioctadecyl-3,3,3′,3′-tetramethylindo-carbocyanine perchlorate (DiI) fluorescent dye following the standard protocols as described in previous study [31]. Briefly, 4 mL of 0.5 mM (5.8 mg/L) PNC was mixed with 200 μL Dil dye solution [0.3 mg/L, in dimethylsulfoxide (DMSO)] at 1000 rpm continuous stirring for 1 min at ambient temperature. The obtained mixture was then purified at 4500 rpm for five cycles (5 min each cycle) using a 10 K Amicon cell (MWCO 30 K, Millipore Inc.). Eventually, final concentration of Dil-PNC solution was calculated using the same method as described in previous section (Additional file 1: Figure S1).

Seeds of *Brassica napus* were immersed in the Dil-PNC + TES solution for the 1, 3, and 8 h with TES (10 mM) as a control group. At 1 h, 3 h and 8 h priming, the seeds were washed with DI water and dried with blotted papers in order to prevent the dragging of priming solution from the seed coat to cotyledon and radicle. Using a super thin double edge razor, the seeds were immediately sliced into seed coat, cotyledon, and radicle. The details of the procedure are: in the dark environment the seeds were held in forceps and were sliced into seed coat, cotyledon, and radicle (~ 200 µm size) with the help of super thin double edge razor. Immediately, the sliced samples were mounted on slides. A drop of perfluorodecalin (PFD) was applied to each slice for better quality of confocal imaging. A square coverslip was placed on the mounted sample and was gently pressed to well-cover the sample with observation gel and to remove the air bubbles trapped underneath. The prepared sample slide was placed on the microscope and imaged by a Leica laser scanning confocal microscope (TCS, SP8). The imaging settings are as follows: 514 nm laser excitation; Z-Stack section thickness: 4 µm; PMT1, 550–615 nm, for DiI-PNC fluorescence; PMT2, 700–750 nm, for the possible fluorescence of different seed tissues. 3–4 seeds were used for confocal imaging.

### Measurement of α-amylase activity, water content, and total soluble sugar content in rapeseeds

For the imbibition experiments, 0.5 g seeds were immersed in the respective solutions described in previous section. The water absorbance by the seeds was calculated from difference in initial weight and the water absorbed during different priming hours. The activity of α-amylase was determined by 3, 5-dinitrosalicylic acid (DNS) method [33]. For each treatment, 1 g seeds was ground in liquid nitrogen with the help of mortar and pestle. The grinded samples were transferred into centrifuge tubes containing 10 mL of phosphate buffer (pH 7). Pipetted 100 µL enzyme solution into tubes containing 1 mL DNS reagents (3, 5-dinitrosalicylic acid (0.4 g), sodium potassium tartrate (1.82 g), phenol (0.02 g), sodium sulphate (0.5 g), and sodium hydroxide (0.1 g) were mixed in 1000 mL ddH_2_O) and 1 mL distilled water. All the tubes were incubated in hot water bath for 10 min, and then were allowed to cool down at ambient temperature. Finally, the samples were well-mixed and the absorbance was recorded at 540 nm while tarring the UV–Vis spectrophotometer (UV 1800PC, AOE, Shanghai, China) with a blank sample. The rate by which maltose is liberated from starch was measured by its ability to reduce 3, 5-dinitrosalicylic acid. For the standard curve, different concentrations of maltose (0.5, 1, 1.5, 2, 2.5, 3, and 3.5 µM) were used. The activity of amylase was measured according to calculation method: α-amylase (mg g^−1^ min^−1^) = Absorbance × standard factor × dilution)/(time of incubation × Mol. wt of maltose) [34]. The total soluble sugar content was determined by following the instructions provided by Suzhou Biotechnology Co., Ltd. “Kit for total soluble sugar determination” (item number: G0501W).

### Quantification of ROS levels and antioxidants enzymes assay

After 7 days of sowing seeds in salt or normal growing conditions, seedlings in the germination box were washed with ddH_2_O, surface dried with blotted papers and immediately dissected into roots and shoots. After weighing ~ 0.1 g root and shoot, the samples were immediately stored into a tank containing liquid nitrogen and then transferred into −80 ℃ refrigerator until further use. Three biological replicates (one batch containing 50 seedlings as one biological replicates) were used for the quantification of ROS level and activities of antioxidant enzymes. The readings were recorded using UV–Vis spectrophotometer (UV 1800PC, AOE, Shanghai, China).

For the determination of hydrogen peroxide (H_2_O_2_) and superoxide anion (^•^O_2_^−^) assay, kits from “Nanjing Jiancheng Biotechnology Co., Ltd (item number: A04-1-1)” and “Beijing Leigen Biotechnology Co., Ltd. (item number: TO1123)” were used respectively. H_2_O_2_ and ^•^O_2_^—^ contents were measured according to the instructions provided by the manufacturer. Malondialdehyde (MDA) content was determined by homogenizing 0.1 g fresh sample with 1 mL of 5% (w/v) trichloroacetic acid at 10,000 g for 10 min [34]. Subsequently, 0.4 mL of 5% of trichloroacetic acid (TTCA) containing 0.67% (w/v) thiobarbituric acid (TBA) was added to 0.4 mL of the supernatant. The absorbance was recorded via spectrophotometer at 450 nm, 532 nm, and 600 nm. MDA was quantified as follow: MDA concentration (μmol/L) = 6.45 (A_532_-A_600_)-0.56A_450_, where A = absorbance at different wavelengths. Finally, MDA content (µmol/g) = C × V/(1000 × W), C = concentration of MDA, V = sample extraction liquid (mL), W = weight of sample.

The activities of superoxide dismutase (SOD) was measured by homogenizing 0.1 g fresh sample in 1 mL of phosphate buffer (pH 7.8) having 0.1 mM EDTA [35]. The homogenate was centrifuged at 12,000 rpm at 4 °C. In a 10 mL tube, 0.2 ml of the enzyme extract, 0.3 mL of 130 mmol/L Met (methionine), 0.3 mL of 750 µmol/L NBT (nitroblue tetrazolium), 0.3 mL of 100 µmol/L EDTA-Na_2_, and 0.3 ml of 20 µmol/L flavin were added. At the end of the reaction, dark control tube was used as a blank control, and the absorbance was recorded at 560 nm using spectrophotometer. SOD activity (U/g) = 2(ACK-AE) × V/(ACK × W × Vt), ACK = dark control absorbance, AE = sample absorbance, V = volume of sample (mL), W = weight of sample (g), Vt = extract liquid volume (mL). The catalase (CAT) activity was measured according to the standard procedures of previous publication by Chakraborty et al. [36]. Samples were ground with PBS (phosphate-buffered saline) buffer (0.2 mol/L Na_2_HPO_4_, 0.2 mol/L NaH_2_PO_4_·H_2_O, pH 7.8) followed by centrifugation at 4000 rpm (15 min). After the centrifugation, the collected supernatant was vortexed with PBS buffer (pH 7.8) and H_2_O_2_ (10 mM). At 240 nm (1 record/1 min, 4 min), the average decrease in the absorbance was recorded and the absorbance coefficient of 43.6 M^−1^ cm^−1^ was used. The final value of CAT was expressed as mmol H_2_O_2_/mg protein/min. Peroxidase (POD) activity was determined using guaiacol method [34]. The reaction mixture was comprised of 10 mM guaiacol, 5 mM H_2_O_2_ in 50 mM phosphate buffer (pH 7.0) incubated at 25 °C. In 10 mL tube, 0.2 mL enzyme extract and 2.8 mL of the reaction mixture was added and mixed subsequently while the absorbance was recorded at 470 nm. Using the absorbance coefficient of 26.6 mM^−1^ cm^−1^, the POD activity was calculated via analyzing the averaged decrease of the recorded absorbance value at 470 nm (1 record/1 min, 3 min). The final value of POD was expressed as μmol tetra-guaiacol /mg protein /min.

### Estimation of sodium (Na^+^) and potassium (K^+^) content

For measuring Na^+^ and K^+^ content, the seedlings in the germination box were washed with ddH_2_O to remove the NaCl solution adhered to the seedlings. Then, seedlings were blotted with tissue paper to remove the water and separated into roots and shoots. Fresh weight was recorded immediately. Samples were then kept in the oven (70 °C, 72 h) for drying. Dry weight was also recorded. About 0.05 g dried samples were ground with a grinder, and then transferred into a 50 mL glass tube and added with 0.2 mL of ddH_2_O. The samples were digested for 1.5 h in 5 mL of concentrated H_2_SO_4_ (18.4 M). Then, 0.2 mL 30% H_2_O_2_ was added and mixed for 30 min. Once the white smokes appeared, samples were taken out from the digestion instrument (LWY84B, Siping Institute of Electronics Technique, China). After cooling down to room temperature, ddH_2_O was added to make the digested solution to 50 mL. Flame photometer (FP6431, Jiangke, Shanghai, China) was used to determine the content of K^+^ and Na^+^ in the samples. The standard curve for K^+^ and Na^+^ were set up according the protocols for running the flame photometer model no. FP6431 [37].

### RNA isolation and quantitative real-time PCR (qRT-PCR) analysis

RNAprep Pure Plant Kit (RN38, Aidlab, Beijing, China) was used for the total RNA isolation. Using the TRUEscript first Strand cDNA Synthesis Kit (PC5402, Aidlab, Beijing, China), 2 μg of total RNA was reverse transcribed into cDNA. According to the manufacturer’s instructions, the amplification of qRT-PCR products was performed in a reaction mixture of 12.5 μL SYBR Green qPCR Mix (PC3302, Aidlab, Beijing, China). The qRT-PCR analysis was performed on the Bio-Rad CFX Connect Real-Time PCR System (Bio-Rad, California, USA). For each treatment, three technical and three biological replicates were used. Relative gene expression was calculated using the 2^−ΔΔCt^ method. The primers used for qRT-PCR are shown in Additional file 1: Table S1 [38].

### Statistical analysis

Means were compared using Independent-Samples T Test or One-Way ANOVA based on Tukey or Duncan test in SPSS software. All data were subjected to normal distribution tests by using non-parametric tests based on 1-Sample K-S (Kolmogorov–Smirnov test). * represents *p* < 0.05. Different lowercase alphabets indicate significant difference at *P* = 0.05. Error bars are standard error. The graphs were plotted with Excel 2016.

## Results

### Characterization of PNC

**Fig. 1:**
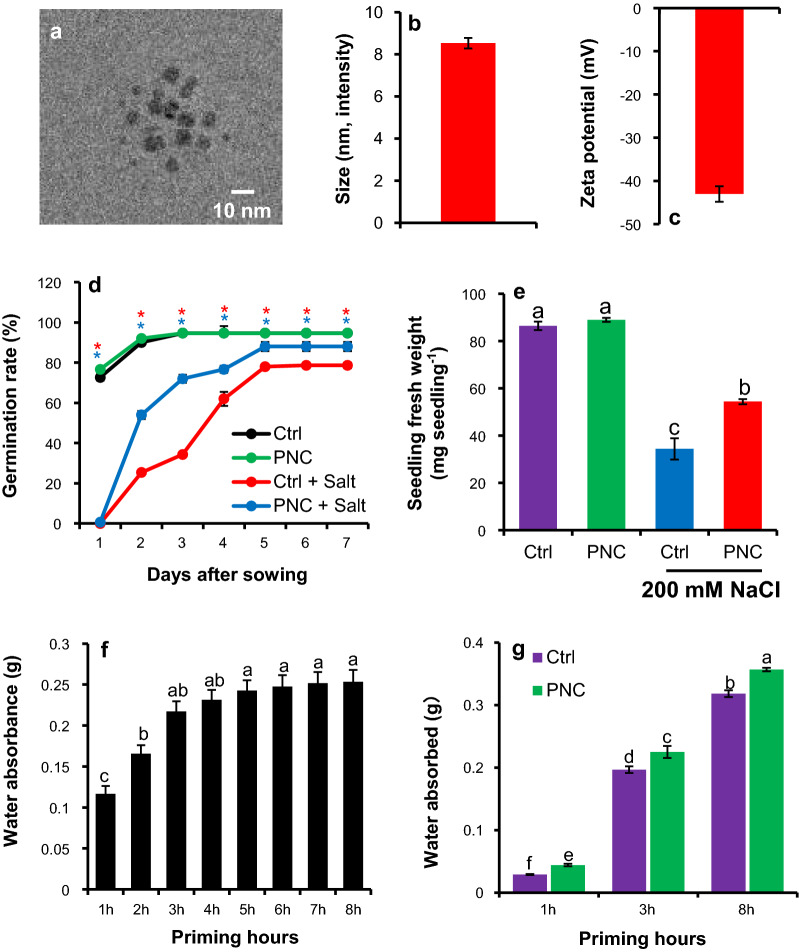
Characterization of cerium oxide nanoparticles (PNC) and the effect of PNC nanopriming on seed germination and fresh weight of rapeseed under salt stress. **a** TEM image of PNC, **b** PNC size by intensity, **c** zeta potential of PNC, **d** and **e** the effect of PNC priming on rapeseed germination rate and fresh weight of seedlings under salt stress or non-saline condition, **f** water uptake by seed from 1 to 8 priming hours with an interval of 1 h, **g** comparison of absorbed water content between PNC priming and control during 1 h, 3 h, and 8 h. Different lowercase alphabets on the vertical bars or * indicates significant difference at *P* < 0.05. Error bars are the representative of standard error of three biological replicates (one batch as one biological replicates) (n = 3)

A clear peak at 271 nm was observed in the absorbance curve of PNC (Additional file 1: Figure S1). The images from transmission electron microscopy (TEM) shows an average PNC core size of 4.7 ± 0.9 nm (Fig. 1a). Analysis from a dynamic light scattering instrument (Malvern Zetasizer, Nano) showed that the average size of PNC by intensity was 8.5 ± 0.2 nm (Fig. 1b), and the average zeta potential was −43.3 ± 6.3 mV (Fig. 1c).

### Influence of PNC priming on rapeseed germination and phenotype

The germination rate of rapeseed was not significantly affected by PNC + TES priming under normal growing conditions as compared to TES (or control) priming (Fig. 1d). However, a significant difference of the germination rate was observed between PNC + TES priming and the control/TES priming under 200 mM salinity stress from Day2 to Day7 (trail terminated), showing the final germination rate of 84% and 76% for PNC priming and TES priming (*P* < 0.05), respectively. Compared with TES priming, PNC + TES priming markedly increased fresh weight (54.4 ± 0.3 vs 34.4 ± 0.7 mg/seedling, 41% increase) of *Brassica napus* seedlings under salt stress (Fig. 1e).

### Localization of PNC in seeds during priming hours

**Fig. 2:**
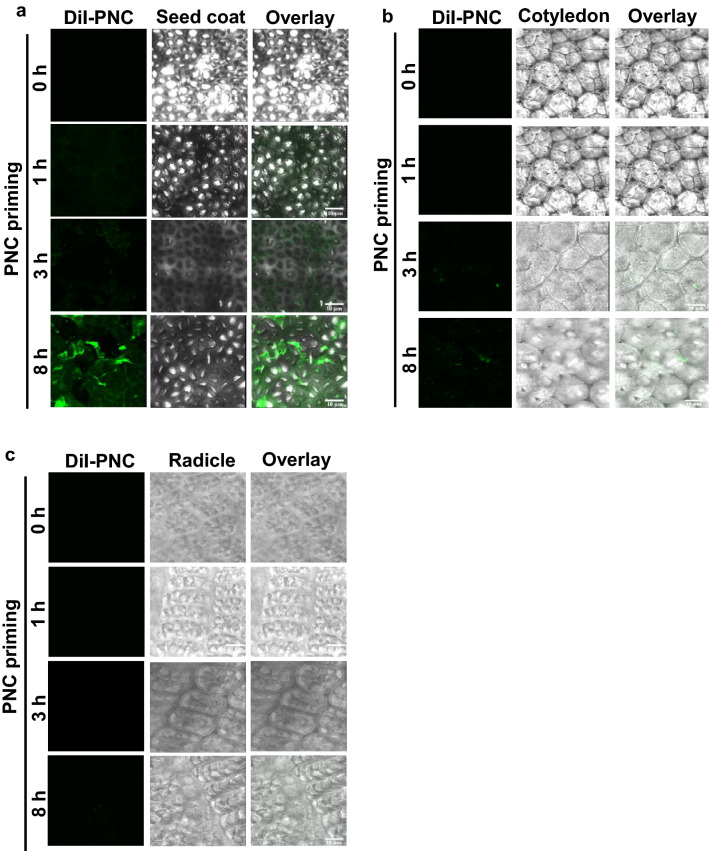
Confocal imaging of Dil-PNC distribution in seed coat, cotyledon, and radicle of the PNC primed seeds. DiI-PNC signal in seed coat (**a**), cotyledon (**b**), and radicle (**c**) of rapeseed with PNC priming at 1 h, 3 h, and 8 h of priming. Scale bar: 10 µm

A clear increase of water content was observed in seeds of *Brassica napus* during priming hours, showing a sharp increase from 1 to 3 h (41% to 99% increase in water uptake), and a steady increase from 4 to 8 h (107% to 118% increase in water uptake) (*p* < 0.05) (Fig. 1f). The sampling hours (1 h, 3 h, and 8 h) were based on a preliminary experiment in which we measured the water absorbance by seeds. The results indicated that compared to TES priming, PNC primed seeds absorbed more water at the first hour (52% increase) than the 3 h (15% increase) and 8 h (13% increase) (Fig. 1g). Consequently, seeds primed with DiI-PNC in 10 µM TES buffer for 1 h, 3 h, and 8 h were sampled for visualizing the distribution of PNC in seed coat, cotyledon, and radicle. During the first hour of priming, Dil-PNC signal was only detected in the seed coat (Fig. 2a), compared with no Dil-PNC signals were detected in cotyledon (Fig. 2b) and radicle (Fig. 2c). At 3 h priming, Dil-PNC signal was observed in both the seed coat (Fig. 2a) and cotyledon (Fig. 2b), while no Dil-PNC signal was detected in radicle (Fig. 2c). Further, during 8 h priming, Dil-PNC was found in all tissues of the seed, i.e. seed coat, cotyledon, and radicle, showing the signal intensity as seed coat > cotyledon > radicle (Fig. 2a–c). No signals were detected in the control group at 1 h, 3 h, and 8 h priming (Additional file 1: Figure S3–5).

### PNC nanopriming regulated relative expression of *AMY* genes to enhance α-amylase activity and total soluble sugar content in rapeseeds

**Fig. 3:**
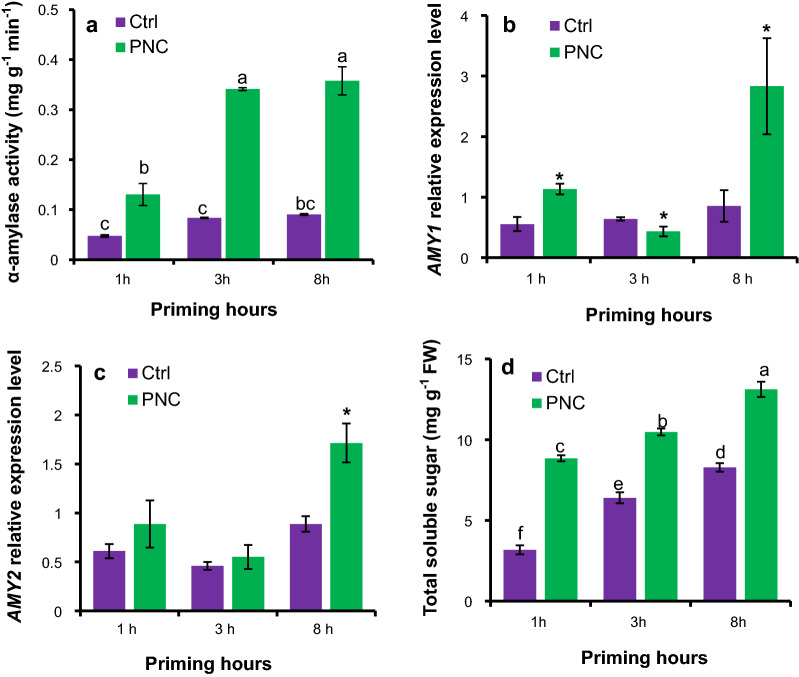
PNC nanopriming modulates the activities of α-amylase in seeds. **a** α-amylase activity in seeds primed with or without PNC at 1, 3, and 8 priming hours, **b** and **c** relative expression level of *AMY1* and *AMY2* genes in seeds primed with or without PNC at 1, 3, and 8 priming hours, and **d** total soluble sugar content in seeds primed with or without PNC at 1, 3, and 8 priming hours. Different lowercase alphabets on the vertical bars or * indicates significant difference at *P* < 0.05. Error bars are the representative of standard error of three biological replicates (one batch as one biological replicates) (n = 3)

Significant differences in α-amylase activity were recorded during the priming hours of PNC priming (*P* < 0.05). Seed priming with PNC + TES increased α-amylase activity by 175%, 309%, and 295% at 1 h (0.130 ± 0.038 vs 0.047 ± 0.003 mg g^−1^ min^−1^), 3 h (0.341 ± 0.005 vs 0.0883 ± 0.001 mg g^−1^ min^−1^), and 8 h (0.357 ± 0.048 vs 0.090 ± 0.002 mg g^−1^ min^−1^) priming, respectively, compared to the TES priming control (Fig. 3a). Consistently, at 1 h and 8 h, the relative expression level for *AMY1* gene was significantly higher in PNC + TES primed seeds than the TES priming control, while a downregulation of *AMY1* gene was found at 3 h (Fig. 3b). The upregulation of *AMY2* gene was found at 8 h priming with PNC + TES than the TES control, compared with no difference at 1 and 3 h priming (Fig. 3c). The highest increase in the relative expression values for *AMY1* and *AMY2* was recorded at 8 h priming, showing 169% and 68% increase respectively (Fig. 3b and c). Compared to TES priming, PNC + TES priming significantly increased total soluble sugar content (TSS) by 177%, 64%, and 58% during 1 h (8.8 ± 0.1 vs 3.2 ± 0.2 mg g^−1^ FW), 3 h (10.4 ± 0.2 vs 6.4 ± 0.2 mg g^−1^ FW), and 8 h (13.1 ± 0.2 vs 8.3 ± 0.2 mg g^−1^ FW), respectively (Fig. 3d). This is in accordance with the increased activities of α-amylase in PNC + TES primed seeds than the TES control.

### PNC nanopriming modulates ROS level in the seeds and seedlings of rapeseeds

**Fig. 4:**
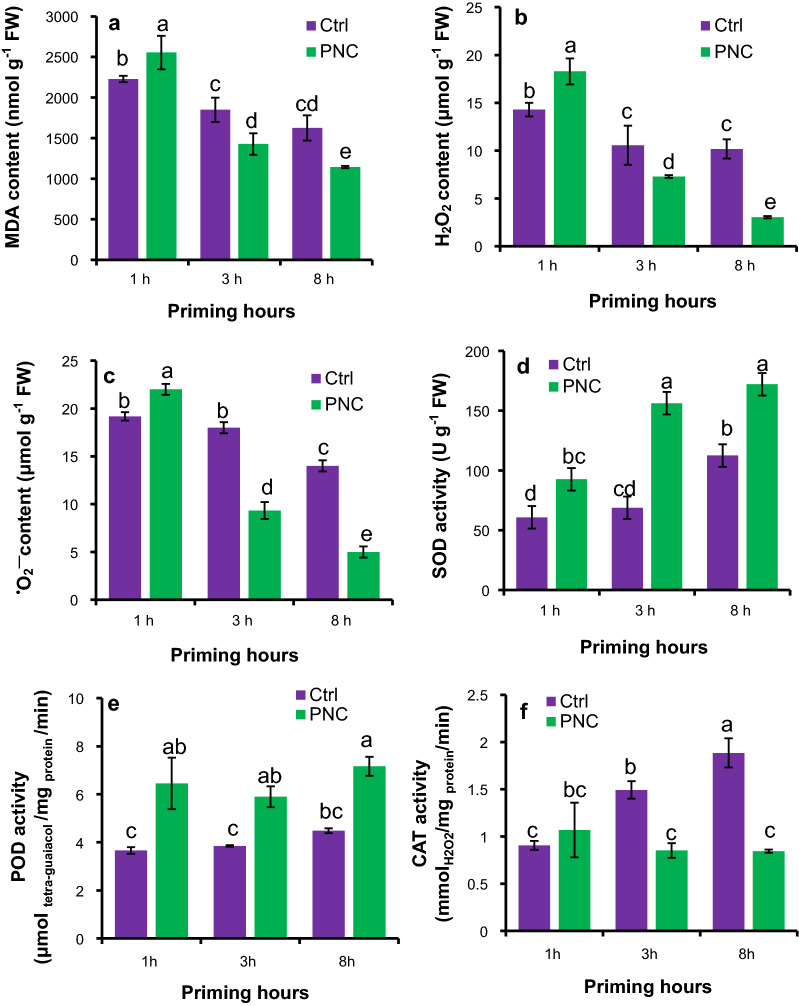
PNC nanopriming modulates ROS homeostasis and antioxidant enzymes activities in seeds. MDA content (**a**), H_2_O_2_ content (**b**), ^•^O_2_^−^ content (**c**), SOD activity (**d**), POD activity (**e**), and CAT activity (**f**) in seeds primed with or without PNC at 1, 3, and 8 priming hours. Different lowercase alphabets on the vertical bars indicates significant difference at *P* < 0.05. Error bars are the representative of standard error of three biological replicates (one batch as one biological replicates) (n = 3)

At 1 h, compared to TES priming, PNC priming showed significantly increased MDA contents (2555 ± 206 mg g^−1^ FW), H_2_O_2_ (18.2 ± 1.3 μmol g^−1^ FW), and ^•^O_2_^−^ (22.3 ± 0.3 μmol g^−1^ FW) by 15%, 28%, and 15%, respectively (Fig. 4a–c). Whereas at 3 h and 8 h, PNC priming significantly reduced MDA contents (1428 ± 156 and 1144 ± 14 mg g^−1^ FW for 3 h and 8 h, 23% and 30% decrease respectively), H_2_O_2_ (7.3 ± 0.1 and 3.0 ± 0.1 μmol g^−1^ FW for 3 h and 8 h, 31% and 70% decrease respectively), and ^•^O_2_^−^ content (9.3 ± 0.4 and 5.2 ± 0.2 μmol g^−1^ FW for 3 h and 8 h, 48% and 64% decrease respectively) than the TES priming (Fig. 4a–c). A significant increase of SOD and POD activities was found in seeds primed with PNC + TES than the TES control (Fig. 4d and e). PNC priming increased SOD activities in seeds than the control by 52%, 127%, and 53% at 1 h (92.6 ± 1.3 U g^−1^ FW), 3 h (156.2 ± 1.3 U g^−1^ FW), and 8 h (172.1 ± 1.3 U g^−1^ FW), respectively (Fig. 4d). PNC priming increased POD activities in seeds than the control by 76% and 53% and 60% at 1 h (6.4 ± 1.0 μmol tetra-guaiacol /mg protein /min), 3 h (5.9 ± 0.4 μmol tetra-guaiacol /mg protein /min) and 8 h (7.1 ± 0.3 μmol tetra-guaiacol/mg protein/min), respectively (Fig. 4e). In contrast to the changes of SOD and POD activities, PNC priming resulted significantly lower CAT activity in seeds than the control, showing 43% and 55% decrease at 3 h (0.85 ± 0.07 mmol H_2_O_2_/mg protein/min), and 8 h (0.84 ± 0.07 mmol H_2_O_2_/mg protein/min), respectively (Fig. 4f).

**Fig. 5:**
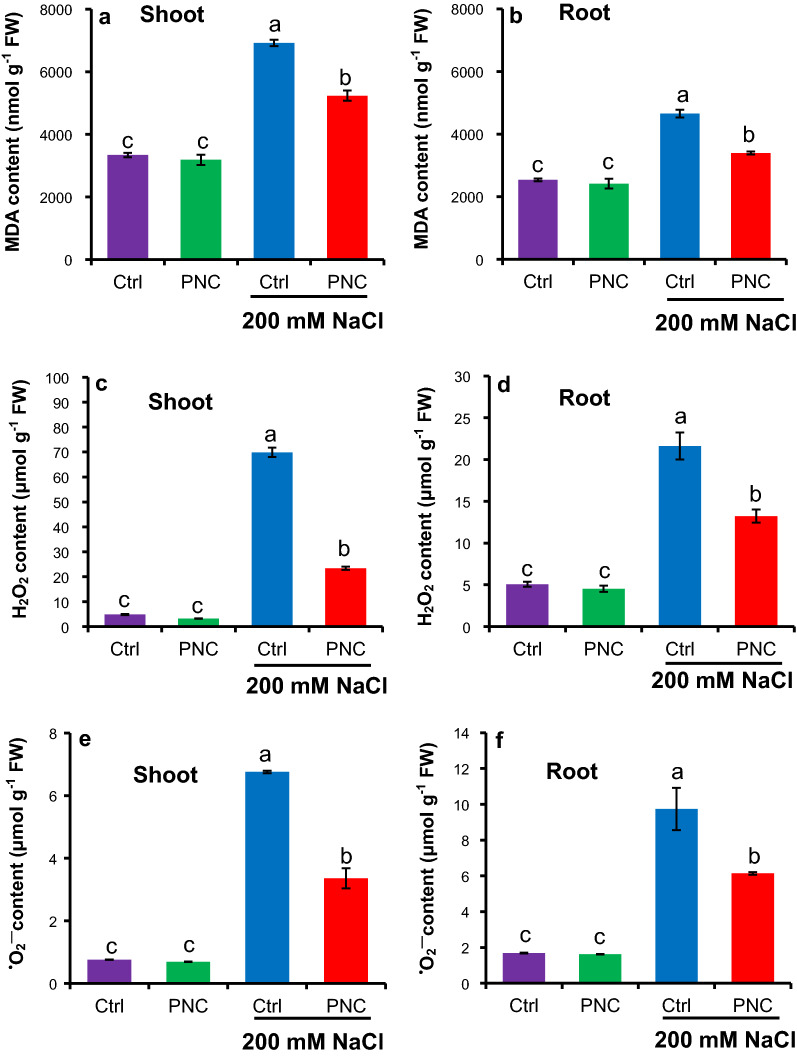
PNC nanopriming modulates ROS level in salt stressed seedlings established from the primed seeds. MDA content (**a** for shoot,** b** for root), H_2_O_2_ content (**c** for shoot, **d** for root), and ^•^O_2_^—^ content (**e** for shoot,** f** for root) in seedlings established from seeds primed with or without PNC under 200 mM NaCl stress (7 days) or non-saline condition. Different lowercase alphabets on the vertical bars indicates significant difference at *P* < 0.05. Error bars are the representative of standard error of three biological replicates (one batch as one biological replicates) (n = 3)

**Fig. 6:**
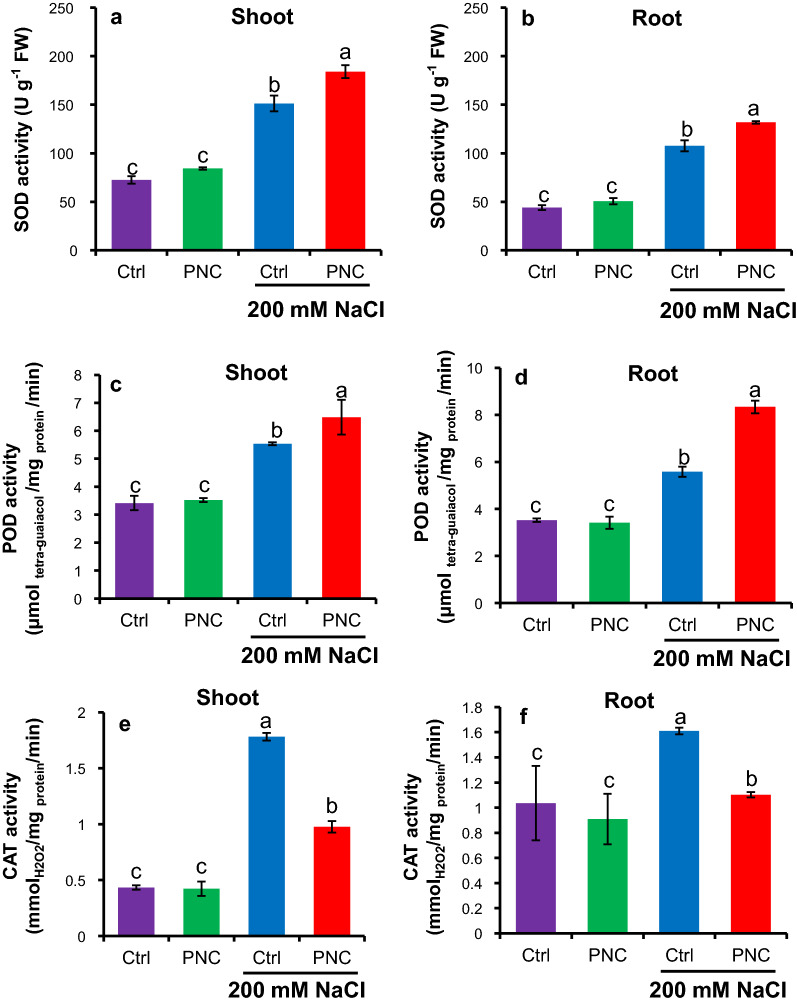
PNC nanopriming modulates antioxidant enzyme activities in salt stressed seedlings established from the primed seeds. Activities of SOD enzyme (**a** for shoot,** b** for root), POD enzyme (**c** for shoot, **d** for root), and CAT enzyme (**e** for shoot,** f** for root) in seedlings established from seeds primed with or without PNC under 200 mM NaCl stress (7 days) or non-saline condition. Different lowercase alphabets on the vertical bars indicates significant difference. Error bars are the representative of standard error of three biological replicates (one batch as one biological replicates) (n = 3)

Compared with non-stress condition, MDA, H_2_O_2_, and ^•^O_2_^−^ contents were significantly increased in salt stressed (200 mM NaCl, 7 days) seedlings of rapeseeds with or without PNC priming (Fig. 5a–e). Under non-saline condition, no difference of MDA, H_2_O_2_, and ^•^O_2_^−^ content was found in either shoot or root seedlings of rapeseeds (7 days old) with or without PNC priming. However, under salt stress, PNC primed seedlings significantly reduced MDA (5234 ± 165 mg g^−1^ FW, 24% decrease), H_2_O_2_ (23.4 ± 0.6 μmol g^−1^ FW, 66% decrease), and ^•^O_2_^−^ (3.4 ± 0.3 vs 6.7 ± 0.3 μmol g^−1^ FW, 50% decrease) content in shoot than the control (Fig. 5a, c and e). Similarly, compared with the control, 27%, 39%, and 37% decrease of MDA (3395 ± 47 mg g^−1^ FW), H_2_O_2_ (13.2 ± 0.7 μmol g^−1^ FW), and ^•^O_2_^−^ (6.1 ± 0.7 μmol g^−1^ FW) content was found in the root of PNC primed seedlings (Fig. 5b, d and f). Similar to the results of the seeds during priming hours, compared with a decrease in CAT activities (0.94 ± 0.05 μmol H_2_O_2_/mg protein/min for shoot, and 1.10 ± 0.02 μmol H_2_O_2_/mg protein/min for root), a significant increase of SOD (183.9 ± 6.6 U g^−1^ FW for shoot, and 131.7 ± 1.2 U g^−1^ FW for root) and POD (6.5 ± 0.6 μmol tetra-guaiacol/mg protein/min for shoot, and 8.8 ± 0.3 vs 5.6 ± 0.2 μmol tetra-guaiacol/mg protein/min for root) activities was found in shoot and root of seedlings primed with PNC + TES than the TES control under salt stress (Fig. 6a–e). Under non-saline condition, no significant difference was found between seedlings primed with and without PNC priming (Fig. 6a–e). It shows that PNC priming enhanced shoot and root SOD (21% and 22%, respectively) and POD (17% and 49%, respectively) activities in contrast to the control under salt stress conditions (Fig. 6a–d), while a decrease of CAT activity by 82% and 31% was observed in the shoot and root compared to the control under salt stress (Fig. 6e–f).

### PNC nanopriming affected Na^+^/K^+^ ratio in rapeseed under salt stress

**Fig. 7:**
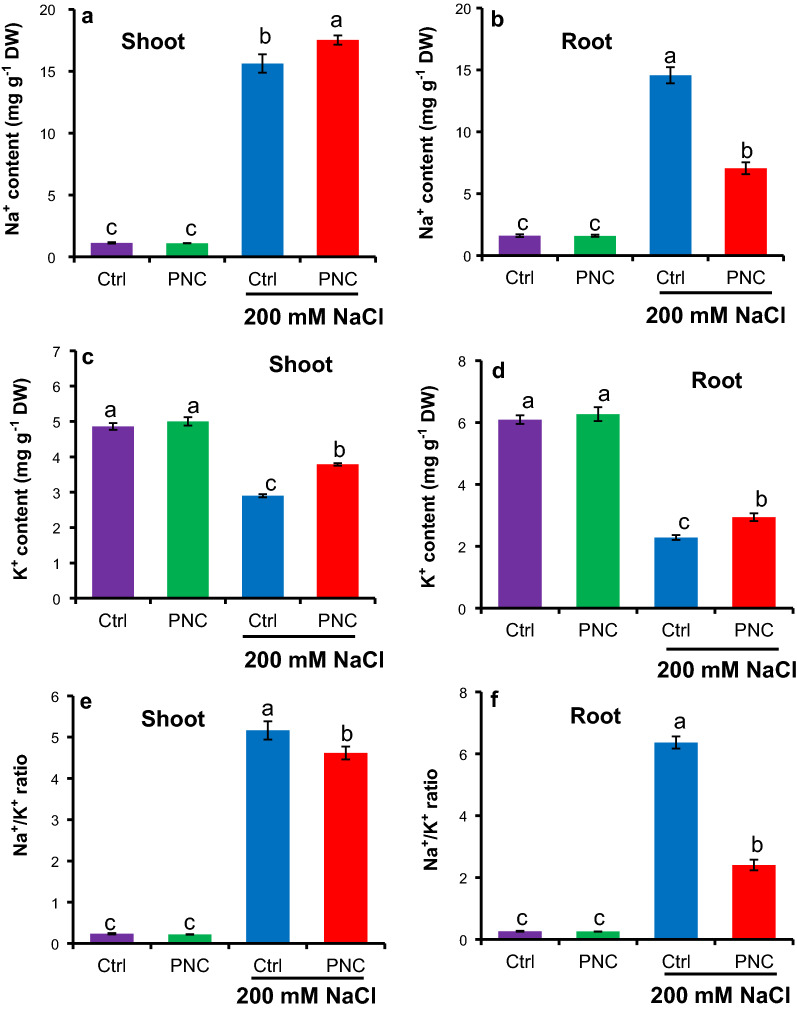
PNC nanopriming modulates Na^+^ and K^+^ content and Na^+^/K^+^ ratio in rapeseed shoot and root under salt stress. Na^+^ content (**a** for shoot,** b** for root), K^+^ content (**c** for shoot, **d** for root), and Na^+^/K^+^ ratio (**e** for shoot,** f** for root) in seedlings established from seeds primed with or without PNC under 200 mM NaCl stress (7 days) or non-saline condition. Different lowercase alphabets on the vertical bars indicates significant difference at *P* < 0.05. Error bars are the representative of standard error of three biological replicates (one batch as one biological replicates) (n = 3)

Compared to non-saline conditions, an increase of Na^+^ content was found in salt stressed (200 mM NaCl, 7 days) seedlings of rapeseeds with or without PNC priming (Fig. 7a–b). However, seedlings with PNC priming showed increased shoot Na^+^ content by 13% (17.5 ± 0.4 vs 15.6 ± 0.7 mg g^−1^ DW, Fig. 7a) and decreased root Na^+^ content by 52% (7.0 ± 0.5 vs 14.5 ± 0.6 mg g^−1^ DW, Fig. 7b) than the control under salt stress. An overall decrease of Na^+^ content was observed in seedlings primed with PNC than the control under salt stress (24.5 ± 0.6 vs 30.8 ± 0.6 mg g^−1^ DW, Additional file 1: Figure S6a). In comparison with non-saline conditions, K^+^ content in shoot and root of seedlings with or without PNC priming was significantly reduced under salt stress (200 mM NaCl, 7 days) (Fig. 7c and d). While seedlings primed with PNC maintained higher K^+^ content in the shoot and root by 31% (3.80 ± 0.06 vs 2.91 ± 0.27 mg g^−1^ DW, Fig. 7c) and 29% (2.9 ± 0.2 vs 2.3 ± 0.1 mg g^−1^ DW, Fig. 7d), respectively, than the control (seedlings primed with TES) under salt stress. An overall better maintained K^+^ content was observed in seedlings primed with PNC than the control under salt stress (6.7 ± 0.2 vs 5.1 ± 0.2 mg g^−1^ DW, Additional file 1: Figure S6b). Not surprisingly, compared to the TES control, seedlings primed with PNC showed significantly reduced Na^+^/K^+^ ratio by 10% and 62% in the shoot (4.6 ± 0.2 vs 5.1 ± 0.2, Fig. 7e) and root (2.4 ± 0.2 vs 6.3 ± 0.2, Fig. 7f) under salinity stress, respectively. An overall decreased Na^+^/K^+^ ratio was observed in seedlings primed with PNC than the control under salt stress (3.65 ± 0.07 vs 5.81 ± 0.08, Additional file 1: Figure S6c). No significant difference of Na^+^/K^+^ ratio in either shoot or root was found in seedlings primed with and without PNC under non-saline condition (Fig. 7e and f).

## Discussion

### PNC nanopriming improves rapeseed salt tolerance through modulating α-amylase activity

In terms of nano-enabled agriculture, nanopriming technique could be a good candidate to improve plant tolerance to salinity stress [39]. Salinity is known to delay seed germination and to limit seed germination rate [2, 40]. Rapeseed is a moderate salt tolerant species [41, 42]. In this study, we recorded that in non-primed group, the germination rate in non-saline condition reached to plateau stage at day2 (95%) whereas it takes 5 days to reach the plateau (76%) under saline condition. Under salinity stress, priming seeds with PNC helped to improve seed germination rate to 84%, but still requires five days to reach the plateau (Fig. 1d). Also, under salinity stress, PNC priming increased the seedling fresh weight (41%) in rapeseed than the TES buffer group (Fig. 1e). Our results showed that PNC nanopriming significantly improved rapeseed tolerance to salinity stress. Similarly, previous studies showed that priming seeds with cerium oxide nanoparticles improved biomass in cotton [27]. Furthermore, under salinity stress, the biggest difference of seed germination rate between PNC priming (72%) and non-priming (34%) groups was observed at day 3 (Fig. 1d). Whereas, under salinity stress, priming cotton seeds with cerium oxide nanoparticles does not result in improvement of germination rate [27]. Under 150 mM NaCl, after 8 h priming (same as in the current study), the highest difference of seed germination rate in lettuce (*Lactuca sativa* variety Parris Island) was found between non-primed seeds and seeds primed with carbon nanoparticles at day 9 [43]. It suggests that in terms of the germination time and seed germination rate, factors such as plant species and types of nanoparticles affect the outcome of seed nanopriming.

In wheat, seed priming with silver nanoparticles (AgNPs) improved the germination and growth parameters by regulating hormonal balance and photosynthetic efficiency under salt stress [44]. Under salt stress, nanopriming with calcium silicate (Ca_2_SiO_4_) in lettuce improved seed germination through triggering antioxidant enzymes including SOD, CAT and GR (glutathione reductase) to effectively scavenge the over-produced ROS [45]. Water soluble carbon nanoparticles (CNPs) promoted lettuce germination and lateral root growth under salt stress [43]. Titanium dioxide (TiO_2_) nanopriming positively affected germination and seedling growth under salt stress by promoting antioxidant enzymes, relative water content, proline content, K^+^ content, and reduced Na^+^ content in maize crop [26]. Compared with the water control, soaking cotton seeds with nanoceria showed increased tolerance (46% increase of fresh weight) to salt stress, whereas no significant difference of germination rate was found between the treatments [27]. During the imbibition of water, nanoparticles enters the seed via the void spaces in the seed coat while some nanoparticles may also tend to adsorb at the surface of seed coat [46]. However, the surface adsorptions of the nanoparticles at seed coat may pose negative effects on germination, seedling growth, and enzymatic activities [47]. Here, we show that during priming hours, most of PNC is located at the surface of seed coat (Fig. 2), indicating a possible interaction between PNC and seed coat which might benefit seed performance under salt stress.

Generally, seed germination begins with imbibition (water uptake by seed) and terminates with the protrusion of radicle and plumule through the seed envelope [48]. During the germination, α-amylase is the key enzyme responsible for the degradation of starch [49]. Thus, under stress conditions the enhancement of α-amylase activity during the priming hours is associated with plant stress tolerance [50–52]. The results of our experiment revealed that PNC priming showed increased α-amylase activity than the control during 1 h, 3 h, and 8 h priming hours, which is further confirmed by the upregulation of relative gene expression of *AMY1* and *AMY2* in PNC + TES primed seeds than the TES priming (Fig. 3). Interestingly, we noticed that at 3 h priming, no upregulation of *AMY1* and *AMY2* was observed in PNC primed seeds than the control, while the α-amylase activity is increased in PNC group (Fig. 3), showing that the α-amylase activity could not be fully reflected by the relative expression of α-amylase genes (AMY1 and AMY2 in this study). This could be due to posttranscriptional regulation on α-amylase [53] or some other factors which could affect the activities of α-amylase. Another reason of the enhanced α-amylase activity in PNC + TES primed seeds might be associated with higher uptake of water during the imbibition period (Fig. 1g). During seed priming, higher amount absorbed water is always associated with the increased total soluble sugars [54]. Not surprisingly, significant higher content of total soluble sugars was found in seeds primed with PNC + TES than TES alone, further supporting the higher α-amylase activity in PNC primed seeds (Fig. 3a). This is similar to previous studies showing that nanopriming enhanced α-amylase activity and enhanced soluble sugar content to improve seed germination [49]. The amount of available soluble sugars in seeds are of importance for the following buildup of seedlings and even its stress tolerance [55]. Previous studies showed that nanopriming [Zinc oxide (ZnO-NPs) nanoparticles, 50 ppm, 50 nm, 23 mV] enabled a positive correlation between improved α-amylase activity and seed germination and seedling vigor of lettuce plants [56]. Also, under cadmium stress, compared with control (0 mg/L ZnO NPs), rice plants primed with 25, 50 and 100 mg/L ZnO NPs nanoparticles (30 nm, zeta potential not reported) have significant increase on seedling α-amylase activities and seed germination rate and shoot fresh weight, thus showing improved tolerance to cadmium stress [57]. Researchers found that seeds with higher amount of total soluble sugars showed better performance in seedling buildup and also the tolerance to drought stress [55]. Overall, our results showed that in rapeseeds, PNC nanopriming upregulated the relative expression of *AMY1* gene, showing increased α-amylase activity and thus significant higher total soluble sugar content than the control (TES priming). It then enabled better establishment of seedlings and its tolerance to salinity stress in rapeseed primed with PNC + TES than TES priming. Overall, in accordance with the findings in previous studies [49, 55, 57], our results suggested that improvement of α-amylase activity is one of the mechanisms underlying PNC nanopriming improved salt tolerance in *Brassica napus*.

### PNC nanopriming reduces ROS over-accumulation to maintain better Na^+^/K^+^ ratio homeostasis to improve rapeseed salt tolerance

ROS accumulation is always observed during seed imbibition [58, 59]. Previous studies showed that ROS was accumulated in seeds with nanopriming [46, 48]. For example, AgNPs primed rice and *Vice faba* [48, 60] showed increased ROS content in seeds. While over-accumulation of ROS could induce toxic effects to plants [25, 27]. PNC are known as the potent ROS scavenger [25]. Herein, compared with higher ROS (H_2_O_2_, ^•^O_2_^−^) and MDA content in PNC + TES treated seeds than the TES group during 1 h priming, PNC + TES treatment significantly reduced the over-production of ROS than the TES control in seeds during 3 h and 8 h priming (Fig. 4a–c). The higher ROS content in PNC + TES than TES treatment at 1 h priming could be related to more absorbed water (Fig. 1g) which could trigger ROS accumulation in primed seeds [61, 62]. During priming, cotyledon and radicle are the main sources for the accumulated ROS [58, 63, 64]. While, at 1 h priming, PNC were mainly distributed at the seed coat (Fig. 2a–c), thus it might not be able to scavenge the higher amount of accumulated ROS than the control due to the better water absorbance in PNC primed seeds than the control (Fig. 1g). Previous studies showed that nanopriming with Au-NPs and Ag-NPS in maize [65], rice [30, 48] and *Arabidopsis* [66] increased seed water uptake due to faster imbibition as compared to control group, suggesting that nanopriming increased the transcript level of aquaporin genes (water channels) to regulate enhanced water uptake during germination. The findings of Mahakham et al. [48] suggested the higher water uptake in nanoprimed seeds during the imbibition period resulted more H_2_O_2_ accumulation compared to unprimed seeds. During seed imbibition, one of the major sources of ROS production is the resumption of the respiration of mitochondria [59]. Thus, another possibility of higher ROS in seeds primed with PNC than control at 1 h could be related to the resumption of mitochondrial respiration. This is worthy to be investigated in future studies. At 3 h and 8 h priming, PNC were also found in cotyledon and radicle (Fig. 2a–c), which helped to scavenge more the accumulated ROS in primed seeds. More interestingly, the amount of ROS in seeds are reduced alongside the priming hours, while PNC + TES group showed higher reduced amount of ROS than the TES control (Fig. 4b and c). Maintenance of ROS homeostasis during seed priming is of importance to seed germination and consequent seedling establishment [67]. Our results suggest that PNC nanopriming could enable better ROS scavenging ability in seeds during the priming hours (except the first hour) than the control. This is similar with previous studies [26, 27] showing that maintaining ROS homeostasis is one of the mechanisms underlying nanopriming improved plant salt tolerance.

After 1 h priming, the activities of antioxidant enzymes are associated with ROS level in seeds, showing a significant higher SOD and POD activities in PNC + TES primed seeds than the TES priming at 3 h and 8 h priming (Fig. 4d–e). Similarly, the activity of SOD and POD was reported to increase and thereby controlling the over-production of H_2_O_2_ and ^•^O_2_^—^ radicals in different crops due to nanopriming [68–70]. Nevertheless, in our experiment, PNC reduced the activity of CAT during the priming hours (Fig. 4f). The lower activity of CAT in our experiment could be due to the fact that PNC can mimic CAT activity [24]. These findings are strongly supported by former studies which used spectrofluorometric using the Amplex-Red reagent assay to confirm nanoceria (PNC) catalase-like catalytic activity [71]. Interestingly, the ROS level and the maintenance of ROS homeostasis showed similar trends between the priming hours experiment and the post-germination experiment. PNC priming significantly reduced the over-production of ROS (H_2_O_2_ and ^•^O_2_^−^ radicals) in the shoot and root of seedlings grown under 200 mM salt stress (Fig. 5a–f), suggesting that PNC nanopriming helped to maintain ROS homeostasis in the consequent established seedlings. Also, in contrary to the lower CAT activities, under salinity stress, SOD and POD activities are higher in rapeseed seedlings originated from PNC primed seeds than the control, regardless of shoot or root (Fig. 6a–f), showing that PNC nanopriming could affect the efficacy of antioxidant enzyme system in established seedlings. This might be associated with possible epigenetic effects enabled by nanomaterials in seeds [67, 72]. The successful scavenging of ROS by PNC under sub-optimal growing conditions were also reported by several other studies [23, 25, 27, 73]

Besides osmotic stress and ROS over-accumulation, salinity also causes Na^+^ over-accumulation and K^+^ loss in plants [74–77]. The ability of plants to maintain Na^+^ and K^+^ homeostasis is a critical factor for its salt tolerance [78]. As usual, salt stress increased Na^+^ accumulation in shoot and root; however, PNC priming accumulated more Na^+^ in shoot while less in the root (Fig. 7a–b). This is in accordance with previous study which showed that CeO_2_ NPs allowed more Na^+^ to accumulate in shoot compared to root by decreasing root apoplastic barriers to facilitate Na^+^ transportation to shoot to improve rapeseed salt tolerance [23]. The overall Na^+^ content in seedlings established from PNC primed seeds is significantly lower than the control under salt stress (Additional file 1: Figure S6), suggesting that PNC nanopriming could help to reduce Na^+^ over-accumulation in rapeseed under salt stress. This is different with the effect of nanoceria nanopriming in cotton, which shows that no significant difference of Na^+^ content in cotyledon, hypocotyl, and root was found between the seedlings established from nanoceria primed seeds and the control under salt stress [27]. Furthermore, compared with the non-saline condition, salt stress decreased K^+^ content in shoot and root of *Brassica napus* seedlings established with or without PNC nanopriming. Nevertheless, seedlings established from PNC primed seeds showed significantly lower K^+^ loss from the shoot and root than the control under salt stress (Fig. 7c–d), suggesting that PNC nanopriming enabled better K^+^ retention in rapeseed under salt stress. Previous studies showed that higher K^+^ retention is associated with better salt tolerance [25, 27, 79]. In cotton under salt stress, PNC nanopriming resulted in significant lower K^+^ content in root while no difference in cotyledon and hypocotyl [27]. These results suggest that the mechanisms employed in PNC nanopriming improved salt tolerance in rapeseed and cotton are different, indicating the complexity of mechanisms associated with nanopriming improved plant salt tolerance. Moreover, salt stress increased Na^+^/K^+^ ratio in the shoot and root of seedlings established with or without PNC nanopriming, while PNC priming enable a reduced Na^+^/K^+^ ratio in the root and shoot than the control under salt stress (Fig. 7e–f), suggesting that PNC helped to maintain better Na^+^/K^+^ ratio than the control under salt stress. Na^+^/K^+^ ratio is a hallmark of plant salt tolerance [80]. Together with Na^+^ and K^+^ content data, our results suggest that PNC nanopriming could help to maintain Na^+^/K^+^ ratio in rapeseed by reducing Na^+^ over-accumulation and K^+^ loss. Thus, better maintained Na^+^/K^+^ ratio could be another mechanism underlying PNC nanopriming improved salt tolerance in rapeseed.

### Nanopriming could be a promising way to improve plant salt stress tolerance

To improve crop production in lands affected by salinity, different strategies have been practiced [81]. Strategies such as water saving irrigation, drainage system, and soil management practices have been applied to promote agricultural production [82, 83]. However, these approaches are expensive which may cost more money on its implementation. Similarly, approaches i.e. screening and breeding of salt tolerant varieties [84], potential use of halophytes [85], and the use of beneficial soil microorganisms [86] could also promote agricultural production in salt affected soils. While these applications require long time. New approaches such as seed nanopriming [39] which are affordable and not time consuming are encouraged to address salinity issue in agricultural production.

Nanotechnology has potential to provide effective solutions to agriculture-related problems [87, 88]. Since the last couple of decades, significant number of researches have been carried out on application of nanoparticles in agriculture under hostile environmental conditions. For example, to improve salt tolerance in crops, nanoparticles were applied as foliar spray or mixed with soil [89, 90]. Application of cerium oxide nanoparticles improved stress tolerance in *Moldavian balm* (foliar spray application, 50 mg L^−1^, [91]), soybean [addition to dry soil at the rate of 2000 mg L^−1^, [92]], and lettuce (addition to the soil at the rate of 100 mg Kg^−1^ soil, [93]) plants by enhancing antioxidant enzymes activities, osmoregulation, photosynthesis, and water relation. However, foliar or soil application of nanoparticles have some obstacles such as possible high cost and environmental pollution, hindering the adoption of nanotechnology in agriculture and its widespread application. Economic viability and biosafety issues are considered as the major obstacles due to the reason that the higher dosage application of nanoparticles could cost more money and also the application process in agricultural land could lead to serious health and environmental risks [94, 95]. Therefore, nanopriming could be a sustainable strategy which requires a minimum use of nanoparticles by not only reducing the cost due to very small dosage application, but also having less risk of nanomaterials on environment. For the current rapeseeds planting mode in China, estimated seed rate for rapeseed in the field is ~ 5 kg/hectare. For the nanopriming, we need 25 mL (1:5 kg/L) of priming solution for 5000 g seeds. Thus, we need about 0.138 L of PNC (0.1 mM, equal to 11.1 mg/L [24]) for the seed rate/hectare. Thus, the amount of PNC for rapeseed nanopriming (1.5 mg PNC for one hectare) is far less than the soil application (1000 mg Kg^−1^ dry sand and clay mixture, [90] or foliar spray (3400 mg PNC for one hectare, personnel communication to Dr. Lan Zhu at Huazhong Agricultural University) for improving rapeseed salt tolerance. It thus not only reduces the cost, but largely alleviates the concerns about biological effects of nanomaterials in environment.

## Conclusion

In summary, during the priming hours, compared to the seeds primed with TES control solution, uptake of PNC by seeds allows more water absorbance, higher antioxidant enzyme (SOD and POD) activities, lower ROS accumulation, higher α-amylase activity (also the upregulation of *AMY1* gene) and total soluble sugar contents. We also found that during the first 1 h priming, PNC are mainly located at the seed coat. While, at 3 h and 8 h, PNC are gradually absorbed into the cotyledon and the radicle (only observed at 8 h priming). After seedling establishment, PNC nanopriming enabled higher antioxidant enzyme (SOD and POD) activities, lower ROS accumulation, and better maintenance of Na^+^/K^+^ ratio than the TES control under salt stress. Our results add more knowledge to PNC nanopriming improved salt tolerance in *Brassica napus*. Besides the improvement of α-amylase activities and maintenance of ROS homeostasis, the better ability to maintain Na^+^/K^+^ ratio is another mechanism underlying the improved salt tolerance in *Brassica napus* with PNC seed nanopriming. Overall, this could be the first study which investigated the downstream events from nanoparticle distribution, ROS level and antioxidant enzyme activities, and α-amylase activities and its gene expression in seeds during priming hours to the maintenance of ROS homeostasis and Na^+^/K^+^ ratio in salt stressed seedlings primed with nanoceria. However, it remains unclear whether the PNC nanopriming improved salt tolerance could last until plant harvest or not. Field or glasshouse experiments are required in further studies. Whether PNC nanopriming could improve the quality of rapeseed such as oil, protein and fatty acid contents or not is also worthy to be studied in future work.

## Supplementary Information

**Additional file 1: Table S1.** Primers for quantitative real-time PCR (qRT-PCR) analysis. **Figure S1.** Absorbance of PNC and Dil-PNC. A 271 nm peak was observed in PNC, while extra 520 and 560 nm peaks were observed in DiI-PNC. **Figure S2.** Screening of PNC concentration for rapeseed nanopriming. Effect of different PNC concentrations on **a**, rapeseed germination rate at different days and **b**, seedling fresh weight at Day7 under salt stress and non-saline condition. Different lowercase alphabets on the vertical bars indicates significant difference at *P* < 0.05. Error bars are the representative of standard error of three biological replicates (one batch as one biological replicates) (n = 3). **Figure S3.** Confocal imaging of Dil-PNC distribution in seed coat of the control group. DiI-PNC signal in seed coat of rapeseed with TES priming at 1 h, 3 h, and 8 h of priming. Scale bar: 10 µm. **Figure S4.** Confocal imaging of Dil-PNC distribution in seed cotyledon of the control group. DiI-PNC signal in seed cotyledon of rapeseed with TES priming at 1 h, 3 h, and 8 h of priming. Scale bar: 10 µm. **Figure S5.** Confocal imaging of Dil-PNC distribution in seed radicle of the control group. DiI-PNC signal in seed radicle of rapeseed with TES priming at 1 h, 3 h, and 8 h of priming. Scale bar: 10 µm. **Figure S6.** PNC nanopriming modulates Na^+^ and K^+^ content and Na^+^/K^+^ ratio in rapeseeds under salt stress. PNC priming decreased Na^+^ accumulation (**a**), improved K^+^ retention ability (**b**), and reduced Na^+^/K^+^ ratio (**c**) in seedlings under 200 mM NaCl stress (7 days). Different lowercase alphabets on the vertical bars indicates significant difference at *P* < 0.05. Error bars are the representative of standard error of three biological replicates (one batch as one biological replicates) (n = 3).

## Data Availability

The datasets used and/or analysed during the current study are available from the corresponding authors on reasonable request.
